# Relationship Between L4/5 Lumbar Multifidus Cross-Sectional Area Ratio and Fall Risk in Older Adults with Lumbar Spinal Stenosis: A Retrospective Study

**DOI:** 10.3390/geriatrics4020038

**Published:** 2019-06-21

**Authors:** Tadashi Ito, Yoshihito Sakai, Kazunori Yamazaki, Makoto Oikawa, Yoshifumi Morita

**Affiliations:** 1 Three-Dimensional Motion Analysis Room, Aichi Prefectural Mikawa Aoitori Medical and Rehabilitation Center for Developmental Disabilities, Okazaki 444-0002, Japan; 2 Department of Physical Therapy, Graduate School of Medicine, Nagoya University, Nagoya 461-8673, Japan; 3 Department of Orthopedic Surgery, National Center for Geriatrics and Gerontology, Obu 474-8501, Japan; jsakai@ncgg.go.jp; 4 Faculty of Clinical Engineering, School of Health Sciences, Fujita Health University, Toyoake 470-1192, Japan; ymzkk@fujita-hu.ac.jp; 5 Community Rehabilitation Center, Higashi-Hachimantai Hospital, Hachimantai 028-7303, Japan; oidon1105@gmail.com; 6 Department of Computer Science and Engineering, Graduate School of Engineering, Nagoya Institute of Technology, Nagoya 466-8555, Japan; morita@nitech.ac.jp

**Keywords:** fall risk, falls, lumbar multifidus, lumbar spinal stenosis

## Abstract

Various factors, including spinal deformities and trunk muscle atrophy, greatly affect the fall risk among older adults with lumbar spinal stenosis (LSS). However, the etiology of falls in older adults with degenerative LSS and trunk muscle atrophy is poorly understood. We investigated the association between trunk muscle atrophy and falls in older LSS patients. This retrospective study included 99 hospitalized older adults with LSS. Participants completed self-reported fall score questionnaires and were divided into the fall risk (n = 30) and non-fall risk (n = 69) groups. The patients’ low back pain visual analog scale score, Geriatric Depression Scale score, sagittal vertical axis, L4/5 lumbar multifidus cross-sectional area ratio (LMCSAR), and center of pressure (COP) values during quiet standing were evaluated. The fall risk group had a lower L4/5 LMCSAR (*p* = 0.002) and increased COP excursion (*p* = 0.034) than the non-fall risk group. No significant differences were observed in the other measured variables between the two groups. The L4/5 LMCSAR (*p* < 0.001) and COP (*p* = 0.024) were related to fall risk and may be useful in fall risk assessment in such populations. Strategies aimed at enhancing controlled lumbar segmental motion and improving trunk muscle stability or mass may decrease the fall risk in this cohort.

## 1. Introduction

Lumbar spinal stenosis is characterized by diminished available space for the neural and vascular elements in the lumbar spine [1]. It results in the presentation of various symptoms, such as difficulties in standing in an upright position, muscle strength weakness, decreased mobility, and an increased fall risk [2]. Falls are a major risk for fractures in the growing geriatric population in developed countries; they are commonly observed among older adults and are associated with increased morbidity and disability [3]. Approximately 30% of older adults fall each year [4,5]. Many previous studies have reported on several factors such as aging, loss of muscle mass, muscle weakness, musculoskeletal pain, home hazards, and psychotropic medication use that increase fall risk [6,7,8,9]. Spinal kyphosis, which limits the activities of daily living and impairs people’s quality of life, has also been reported to be an important cause of falls [10,11]. Furthermore, other studies have reported that the spine angle is associated with falls among community-dwelling older adults [12,13].

Physical impairments, such as trunk muscle atrophy and spinal deformities, may also lead to an increased risk of falls in older adults with lumbar spinal stenosis. The lumbar multifidus, a deep trunk muscle, is well-known for playing a role in the segmental stability of the lumbar spine [14,15], whereas the erector spinae muscles are essential in ensuring balance when external loads are applied to the trunk [14]. Theoretically, factors such as spinal deformities or trunk muscle atrophy deterioration may also greatly affect the risk of falls in older adults with lumbar spinal stenosis. However, few studies have focused on the association between fall risk and trunk muscle atrophy in this population [14,15]. We hypothesized that trunk muscle atrophy is associated with an increased risk of falls in older adults with lumbar spinal stenosis and aimed to examine this association, so as to aid in the identification of such people who have an increased fall risk.

## 2. Materials and Methods 

### 2.1. Participants

We conducted a retrospective analysis involving patients diagnosed with and conservatively treated for lumbar spinal canal stenosis at the Department of Orthopedic Surgery, National Center for Geriatric and Gerontology in Obu, Japan between November 2012 and November 2018. The diagnosis of lumbar spinal canal stenosis was confirmed using L4/5 area magnetic resonance imaging (MRI) by a spine surgeon. Study participants included hospitalized individuals receiving conservative treatment who had consulted orthopedic surgeons for low back pain (LBP); the participants’ risk of falls was recorded using a simple falls score assessment questionnaire, with a score > 7 indicating a high fall risk [16]. The participants were hospitalized for the administration of the conservative treatment for lumbar spinal stenosis with intermittent claudication and LBP. All experiments were conducted at the authors’ institute. The mean length of the participants’ hospital stay was 2 weeks. The inclusion criteria were: diagnosis of lumbar spinal canal stenosis in the L4/5 area, age older than 65 years, and ability to walk independently without assistive devices. Those with the following characteristics were excluded: presence of severe radicular pain and motor weakness in the lower extremities that affected the standing posture, spinal infection, spinal tumors, vertebral fractures, history of previous back surgery, risk of neuropathic diabetes or uncontrolled diabetes, and cardiovascular, cerebrovascular, or neuromuscular disease. Written informed consent was obtained from each participant prior to study inclusion. All investigations were conducted in accordance with the principles expressed in the Declaration of Helsinki, and the ethics committee of the National Center for Geriatric and Gerontology approved the study (IRB approval number: 586).

### 2.2. Fall Questionnaire Assessment

All participants were evaluated using a simple fall score assessment based on the study by Okochi et al. [16]. The fall score ranges from 0 to 13. The simple fall score was assessed by the following closed-ended (yes or no) questions: (1) Do you have a falls history within the past 12 months? (2) Does your back become bent? (3) Has your gait speed become slower? (4) Do you use a cane? and (5) Do you take five or more different medications every day? A fall score > 7 indicated a high fall risk.

### 2.3. Clinical Evaluation

The L4/5 lumbar multifidus cross-sectional area ratio (L4/5 LMCSAR), sagittal vertical axis (SVA), and stabilometry results were used as clinical evaluation indices. All clinical evaluations were performed by an experienced doctor and a physical therapist [16].

### 2.4. Lumbar Multifidus Cross-Sectional Area

MRI was used to evaluate the cross-sectional areas of the erector spinae and lumbar multifidus muscles at the L4/5 levels. Data extractions were performed using Synapse (Fujifilm Medical, Tokyo, Japan). The participants were placed in a neutral position (supine position, knees extended with the hands across the abdomen) during MRI. The MRI data for the erector spinae muscle and lumbar multifidus cross-sectional areas were assessed by a radiologist. The L4/5 muscle cross-sectional areas were calculated as the erector spinae muscle or lumbar multifidus cross-sectional areas/height^2^. The L4/5 LMCSARs were calculated as: (lumbar multifidus cross-sectional area)/(lumbar multifidus cross-sectional area + erector spinae muscle cross-sectional area) × 100% [17].

### 2.5. Spine Alignment

The SVA assessment is a measure of sagittal alignment [18] and is defined as the horizontal offset from the posterosuperior corner of S1 to the vertebral body of C7. The SVA value was determined by a spine surgeon using Synapse (Fujifilm Medical, Tokyo, Japan).

### 2.6. Balance Assessment

Stabilometry was performed using a Balance Board (Wii, Nintendo, Kyoto, Japan) [19,20,21,22]. All participants stood barefoot for 15 s on the Balance Board with their feet together and eyes closed and were instructed to remain still and relaxed in the standing posture with their arms hanging loosely at their sides. The stabilometry data represented the center of pressure (COP) signals (frequency, 100 Hz) recorded over 15 s using a microcomputer. The mean COP was calculated as follows: *Δ*Y = Y (During) − Y (Pre). Prior to stabilometry assessment by an experienced doctor and a physical therapist, participants were allowed 60 s of practice as a learning attempt. Based on previous studies, the recommended cut-off score for the classification of falls and non-falls was 1.68 for the anteroposterior COP excursion [23].

### 2.7. Low Back Pain Assessment

Pain was assessed using a visual analog scale (VAS) (0 [no pain] to 10 [maximum pain]). Based on the results of the pain severity assessment by Boonstra et al., the LBP severity was defined as 3 or more LBP symptoms [24].

### 2.8. Geriatric Depression Scale

The Japanese version of the Geriatric Depression Scale (GDS-15) was used to assess the number of depressive symptoms (0 to 15) [25]. Higher scores indicate a greater likelihood of depression [25,26]. A score of 7 or higher has been reported as an optimal cut-off point [25]. It was confirmed that there was no correlation between the GDS and fall risk in either group (fall risk group: r = −0.008; non-fall risk group: r = 0.196).

### 2.9. Statistical Analysis

Power analysis was conducted using G*Power (Heinrich Heine University of Düsseldorf, Düsseldorf, Germany) to determine the optimal sample size for a statistical power of 0.8, an alpha value of 0.05, a large effect size (d = 0.8), and an allocation ratio of 0.43. Two-tailed tests were also performed using G*Power. The allocation ratio of 0.43 was based on previous studies [4,5]. All data were analyzed using the Statistical Package for the Social Sciences for Windows version 19.0 (IBM Corp., Armonk, NY, USA). A *p*-value < 0.05 was considered statistically significant. Data are expressed as means ± standard deviations or as median values (range). The normal distribution of variable data was confirmed using the Shapiro-Wilk test. Chi-square tests were used to test for differences in sex and the VAS and GDS scores between the groups. The variable data for individuals in the fall risk and non-fall risk groups were compared using an independent *t*-test (corrected for inequality using Levene’s test) or a Mann-Whitney U test, and logistic regression analysis was used to assess the relationship between fall risk and functional outcomes. A simultaneous logistic regression model was used to determine the odds ratios for L4/5 LMCSAR, COP, and SVA. 

## 3. Results

**Figure 1:**
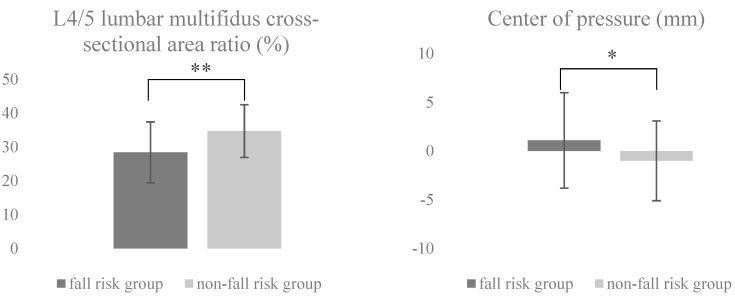
L4/5 lumbar multifidus cross-sectional area ratio for magnetic resonance imaging data and the center of pressure displacement for trials on the balance board. * *p* < 0.05, ** *p* < 0.005.

This study included 99 participants (63 men, 36 women; fall risk group, 30; non-fall risk group, 69) with a mean age of 75.8 (range, 65–85) years who were admitted to the hospital. Each participant was diagnosed with lumbar spinal canal stenosis. The power analysis for the sample size revealed that the optimal sample size for this study was 62 participants (fall risk group = 19 and non-fall risk group = 43). In all, 99 participants (fall risk group = 30 and non-fall risk group = 69) participated in this study. The effect size for the L4/5 LMCSAR was d = 0.8. The effect sizes for the other variables were as follows: COP, d = 0.5; SVA, d = 0.2; age, d = 0.3; height, d = 0.3; weight, d = 0.1; BMI, d = 0.4; sex, Cramer’s V = 0.1; fall score, d = 3.2; VAS, d = 0.3; and GDS, d = 0.3. Table 1 and Table 2 show the baseline clinical characteristics of the study participants and a comparison of the two groups. The fall risk group showed a lower L4/5 LMCSAR (*p* = 0.002) and an increased COP excursion (*p* = 0.034) compared to the non-fall risk group (Figure 1). The age, height, weight, body mass index, sex, VAS score, GDS-15 score, and SVA values were not significantly different. 

Table 3 shows the factors associated with fall risk in the simultaneous logistic regression analysis. Multivariate logistic regression analyses for fall risk, conducted among the significant variables, showed that the L4/5 LMCSAR (*p* < 0.001) and COP (*p* = 0.024) were associated with fall risk (Table 3); therefore, these were determined to be the factors associated with fall risk among the examined trunk function variables. In contrast, the SVA was not associated with the fall risk.

## 4. Discussion

The present study is the first to demonstrate the association between the lumbar multifidus cross-sectional area and the results of the fall risk questionnaire in older adults with lumbar spinal stenosis. Moreover, we demonstrated that there were no significant differences in the SVA between the fall risk and non-fall risk groups. The lumbar multifidus plays an important role in body stabilization [27]. Ward et al. reported that, owing to its relatively short fiber length and large cross-sectional area, the multifidus muscle is uniquely designed as a stabilizer that produces large forces [28]. Thus, in older adults with lumbar spinal stenosis and a high fall risk, the L4/5 lumbar multifidus muscle may not function adequately. Meanwhile, a loss of spinal angle and LBP have been reported as fall risk factors [13,29,30,31,32,33,34,35]. This result conflicts with those of previous reports that demonstrated the association of falls with the loss of spinal angle and LBP [13,29,30,31,32,33,34,35]. In addition, many hospitalized older adults with lumbar spinal stenosis have limited mobility due to advanced spine alignment issues which may weaken the effect of fall risk assessment. Furthermore, this suggests that spine alignment may not be wholly accounted for in fall risk assessment. Marshall et al. reported that, among older men, back pain was related to an increased fall risk [36]. Previous studies have reported that falls in older adults are associated not only with physical functions, such as gait impairment, postural balance impairment, and muscle weakness but also with depressive symptoms [37,38,39]. However, no significant differences were observed in the measured VAS and GDS scores [40,41,42] between the fall risk and non-fall risk groups; many of the hospitalized older adults with lumbar spinal stenosis in both groups may have had comparable depressive and LBP symptoms.

Depression and LBP may not have been wholly accounted for in the fall risk assessment in this study population. Our result indicates that older adults with lumbar spinal stenosis have a small fall risk due to LBP. They also suggest that the GDS-15 was not suitable for the assessment of fall risk in older adults with lumbar spinal stenosis, although it has been reported to be a valid measure of fall risk [38,39]. Therefore, older adults with lumbar spinal stenosis are suggested to have a low fall risk, as indicated by the measures of LBP and the GDS-15. 

The logistic regression analyses showed that the fall risk increased with decreased L4/5 LMCSARs and increased COP excursion. Among the fall risk factors, decreased L4/5 LMCSAR and postural instability associated with lumbar spinal stenosis were identified as being important. Hence, the L4/5 LMCSAR and COP could be useful in the assessment of fall risk in older adults with lumbar spinal stenosis. The maintenance of proper lumbar multifidus function is important in these adults as this muscle affects their ability to perform normal activities associated with daily living. Trunk muscle impairment, the main symptom of lumbar spinal stenosis, results from prolonged hospitalization and leads to muscle weakness and a decreased ability to perform daily activities [43]. Additionally, studies have reported that a higher trunk muscle density is associated with a reduced postural sway in older adults residing in independent living communities [44]. Therefore, the risk of falls among older adults with lumbar spinal stenosis is likely to be affected by postural instability resulting from a decrease in the L4/5 lumbar multifidus size. Consequently, compensation for controlled lumbar segmental motion may be inhibited, further decreasing balance and body stability; any additional decline may induce falls. In addition, trunk muscle impairment may limit the lumbar segmental motion, leading to an increase in the COP excursion. Moreover, a high fall risk can increase the degree of postural instability and sagittal COP values. Zhou et al. reported that an anteroposterior postural sway predicts fall rates in community-dwelling older adults [45]. Thus, compared to those in the non-fall group, older adults with lumbar spinal stenosis with a higher fall risk have postural instability and greater displacement of their sagittal COPs. Previous studies have reported that trunk muscle thickness is decreased in older adults [46]. It is well known that the lumbar multifidus muscles are as important for segmental stability of the lumbar spine as the deep trunk muscles, while the erector spinae muscles are torque generators for spinal motion [47]. Therefore, in the fall risk group, the lumbar multifidus muscles may not be functioning adequately [48] to control postural balance. MRI studies should be used to measure the L4/5 trunk muscle cross-section; however, the associated costs and timing of measurements limit its use in a typical setting of clinical physical therapy. In this regard, ultrasound imaging is an alternate means of assessing the trunk muscle cross-section that could provide insights into the role of trunk muscle function in a typical clinical physical therapy setting [43]. Ultrasound imaging is a non-invasive, easily applied, inexpensive, and practical method used to assess the trunk muscle cross-section in standard clinical practice. Moreover, based on the results of the L4/5 trunk muscle cross-section assessment, fall-risk assessment measures should be utilized in fall-prevention programs for older adults with lumbar spinal stenosis. 

The effect size for the L4/5 LMCSAR was greater than that for the COP excursion. Hence, compared to the COP excursion, the L4/5 LMCSAR may be more useful for the assessment of fall risk in older adults with lumbar spinal stenosis. Thus, it is crucial to assess the L4/5 trunk muscle cross-section to promote fall prevention. In other words, falls may be preventable when the L4/5 LMCSAR and postural stability are improved through exercise therapy, even among older adults with lumbar spinal stenosis.

The present study has several limitations. First, the categorization of fall risk relied on self-reported questionnaire data that could be affected by social desirability bias. Second, the study population included individuals requiring only conservative treatment and who were able to walk independently, possibly leading to the underestimation of the overall fall risk. Third, the assessment of fall risk was limited to a few back muscle impairment measures; the muscle activities and muscle strength of the lumbar multifidus and erector spinae muscles were not evaluated. Trunk muscle impairment associated with aging may lead to the inactivity of the lumbar back muscles, possibly causing pain and changes in the muscle mass, muscle strength, and spinal angle. Fourth, this study had a retrospective design, which did not allow for the prediction of falls in the future. 

## 5. Conclusions

The results of the present study indicated that a decreased L4/5 LMCSAR, more so than an increased COP excursion, may increase the risk of falls in older adults with lumbar spinal stenosis. Therefore, follow-up and longitudinal studies exploring fall prevention strategies to enhance controlled lumbar segmental motion and improve trunk muscle stability or mass are likely to decrease the fall risk in this population. However, the self-reported questionnaire data used in this study could be influenced by social desirability bias. Further longitudinal studies with large sample sizes are needed to identify the mechanisms by which the L4/5 LMCSAR may affect the fall risk in older adults with lumbar spinal stenosis.

## Figures and Tables

**Table 1:** Participant characteristics

Variable	Fall Risk Group	Non-Fall Risk Group	*p*-Value
	(n = 30)	(n = 69)	
Age (years)	77.0 (67.0–96.0)	75.0 (65.0–90.0)	0.178
Height (cm)	155.0 ± 10.1	158.0 ± 8.2	0.125
Weight (kg)	62.1 ± 10.8	61.2 ± 11.0	0.717
BMI (kg/m^2^)	24.7 (19.0–31.2)	23.7 (19.5–31.5)	0.056
Sex (males, %)	16 (53.3)	47 (68.1)	0.160
Fall score (point)	9.0 (8.0–13.0)	4.0 (0–6.0)	0.001
VAS (cm)	5.0 (0–8.0)	4.0 (0–10.0)	0.110
GDS (point)	5.0 (0–14)	5.0 (0–13)	0.643

BMI, body mass index; GDS, Geriatric Depression Scale; VAS, visual analog scale. Data are presented as means ± standard deviations or median values (range). Normal distributions were confirmed using the Shapiro-Wilk test. The sex, VAS, and GDS *p*-values were determined using a chi-square test. The fall score *p*-values were determined using the Mann-Whitney U test. Other *p*-values were generated using an independent *t*-test.

**Table 2:** Demographic characteristics associated with the functional and morphometric outcomes

Variable	Fall Risk Group	Non-Fall Risk Group	*p*-Value
	(n = 30)	(n = 69)	
SVA (mm)	42.9 (−2.1–116.3)	41.3 (−16.9–162.0)	0.493
L4/5 lumbar multifidus cross-sectional area ratio (%)	28.4 ± 9.0	34.7 ± 7.8	0.002
Center of pressure (mm)	1.1 ± 4.9	−1.0 ± 4.1	0.034

SVA, sagittal vertical axis. Data are presented as means ± standard deviations or median values (range). Normal distributions were confirmed using the Shapiro-Wilk test. The SVA *p*-values were measured using the Mann-Whitney U test. Other *p*-values were generated using an independent *t*-test.

**Table 3:** Factors associated with fall risk in the stepwise logistic regression analysis (fall risk group, n = 30; non-fall risk group, n = 69).

Variables	OR	95% CI	*p*-Value
L4/5 lumbar multifidus cross-sectional area ratio	0.911	0.859–0.967	0.001
Center of pressure	1.144	1.018–1.285	0.024
SVA (mm)	1.010	0.994–1.025	0.227

CI, confidence interval; OR, odds ratio; SVA; sagittal vertical axis; The logistic regression analyses indicated that the L4/5 lumbar multifidus cross-sectional area ratio and the center of pressure were independently associated with fall risk. The model was well-calibrated between the observed and expected risk declines. Hosmer-Lemeshow χ^2^ = 9.161, *p* = 0.329.
